# Measurement of non-VKA oral anticoagulants versus classic ones: the appropriate use of hemostasis assays

**DOI:** 10.1186/1477-9560-12-24

**Published:** 2014-11-04

**Authors:** Jonathan Douxfils, Anne Tamigniau, Bernard Chatelain, Catherine Goffinet, Jean-Michel Dogné, François Mullier

**Affiliations:** Department of Pharmacy, Namur Thrombosis and Hemostasis Center (NTHC), Namur Research Institute for LIfe Sciences (NARILIS), University of Namur, Namur, Belgium; Hematology Laboratory, Namur Thrombosis and Hemostasis Center (NTHC), Namur Research Institute for LIfe Sciences (NARILIS), CHU Dinant Godinne UcL Namur, Université Catholique de Louvain, 1, avenue Dr Gaston Therasse, Yvoir, B5530 Belgium; Clinique et Maternité Sainte-Elisabeth, Namur, Belgium

**Keywords:** Vitamin K antagonist, Dabigatran, Rivaroxaban, Apixaban, Low molecular weight heparin, Enoxaparin, Monitoring, Non-VKA oral anticoagulants

## Abstract

Traditional anticoagulant agents such as vitamin K antagonists (VKAs), unfractionated heparin (UFH), low molecular weight heparins (LMWHs) and fondaparinux have been widely used in the prevention and treatment of thromboembolic diseases. However, these agents are associated with limitations, such as the need for regular coagulation monitoring (VKAs and UFH) or a parenteral route of administration (UFH, LMWHs and fondaparinux).

Several non-VKA oral anticoagulants (NOACs) are now widely used in the prevention and treatment of thromboembolic diseases and in stroke prevention in non-valvular atrial fibrillation. Unlike VKAs, NOACs exhibit predictable pharmacokinetics and pharmacodynamics. They are therefore usually given at fixed doses without routine coagulation monitoring. However, in certain patient populations or special clinical circumstances, measurement of drug exposure may be useful, such as in suspected overdose, in patients experiencing a hemorrhagic or thromboembolic event during the treatment’s period, in those with acute renal failure, in patients who require urgent surgery or in case of an invasive procedure. This article aims at providing guidance on laboratory testing of classic anticoagulants and NOACs.

**Electronic supplementary material:**

The online version of this article (doi:10.1186/1477-9560-12-24) contains supplementary material, which is available to authorized users.

## Introduction

Anticoagulants are a mainstay of cardiovascular therapy and, until recently, vitamin K antagonists (VKAs) were the only oral anticoagulants available. The knowledge about monitoring and dosing of VKAs in order to maximize their efficacy and minimize hemorrhagic complications has increased considerably since their introduction in 1950s. In addition, the management of VKAs has been optimized with the establishment of anticoagulation clinics, as well as self-monitoring and self-management programs. However, VKAs have still strong limitations such as a slow onset and offset of action, the requirement of variable dose regimen, a series of multiple drug-drug interactions and a considerable inter-individual variability. These limitations make coagulation monitoring and frequent dose adjustments necessary to ensure an adequate level of anticoagulation. Unfractionated heparin (UFH) and low molecular weight heparins (LMWHs) are also a cornerstone in the armamentarium of anticoagulation. While UFH has to be monitored closely due to unspecified bindings to proteins, endothelial cells and macrophages conducing to a variable response from patient to patient, the interest of monitoring LMWHs is controversial but suggested in specific situations such as in extreme body weights, severe renal insufficiency, pregnancy and cirrhosis.

Non-VKA oral anticoagulants (NOACs) have major pharmacologic advantages over VKAs, including a rapid onset/offset of action, fewer drug interactions, and predictable pharmacokinetics, eliminating theoretically the requirement of regular coagulation monitoring. Regulatory agencies have approved NOACs for various indications based on the results of large phase-III clinical trials demonstrating the efficacy and safety of these compounds. Effectively, they were found at least as efficacious, if not better, than warfarin in the setting of stroke prevention in adult patients with non-valvular atrial fibrillation (NVAF) [1–4] and in the treatment and the secondary prevention of venous thromboembolism [5–9]. Compared to LMWHs, these agents also proved their non-inferiority or superiority for initial treatment of venous thromboembolism and for thromboprophylaxis in patients undergoing hip or knee arthroplasty, [10–20]. Non-VKA oral anticoagulants will certainly replace some of the traditional anticoagulants in the future but it is important to keep in mind that the introduction of these new agents will also change the strategies of patient management and of the hospital routine [21]. Therefore, the knowing of the pharmacology and the impact of these new compounds on routinely used coagulation assays is of great importance to achieve optimal patient outcomes. Moreover, it is anticipated that a non-negligible proportion of patients will reach either insufficient or supra-therapeutic level when given at fixed dose leading to the introduction of dedicated coagulation tests that respond faithfully to the pharmacodynamics of NOACs.

The aim of this review is to define why, when and how to measure traditional anticoagulant and NOACs.

## Pre-treatment biological screening

The following information should be collected before prescribing an anticoagulant at therapeutic or prophylactic dose: cell blood count, prothrombin time (PT), activated partial thromboplastin time (aPTT) and renal function.

Platelet count should be performed before and during follow-up of UFH or LMWH treated patients to screen for immune heparin-induced thrombocytopenia [22, 23].

In clinical trials of oral anticoagulants, drug eligibility and dosing were determined using the Cockcroft-Gault equation to estimate creatinine clearance (CR_CL_) as a measure of renal function. Importantly, it was proved that the use of modification of diet in renal disease (MDRD)-derived estimated glomerular filtration rate (eGFR) instead of Cockcroft-Gault in prescribing anticoagulants leads to overestimation of renal function in lower values [24–26]. Thus, many elderly patients would either incorrectly become eligible for them or would receive a too high a dose.

## Samples acquisition, processing and storage

Sample acquisition and processing are of great importance since it was proven that each component of the specimen collection system (needle gauge, composition of the collecting tube, concentration of sodium citrate) may potentially impact the results for coagulation testing [27]. This should be performed according to international recommendations [27].

For example, contamination of the citrate solution by divalent ion such as magnesium influences PT [28].

For UFH and LMWH monitoring, there is a risk of platelet activation between sampling and centrifugation that leads to neutralization of heparin by binding to PF4 and underestimation of anti-Xa activity. If sampling is performed in citrated tubes, it is important that the delay between sampling and centrifugation is lower than 1 hour and thus to warn the laboratory before sampling. The sample should also be tested within 4 hours [29] The sample may also be collected in a mixture of citrate 109 mM, theophylline, adenosine and dipyridamole (CTAD) which allows increasing the acceptable delay between sampling and centrifugation to 4 hours [30, 31]. Heparin is lost more rapidly in citrate tubes that contain a large air space (after addition of blood) due to accelerated platelet activation and release of PF4. This effect is suppressed if CTAD is used [32].

## Biological monitoring of anticoagulant treatments

### Vitamin K antagonists

Vitamin K antagonists produce their anticoagulant effect by interfering with the cyclic regeneration of vitamin K from the oxidized form to the reduced form. This is achieved by inhibiting the vitamin K epoxyde-reductase. Reduced vitamin K is necessary for the γ-carboxylation of glutamate residues of factors II, VII, IX, X, protein C, S and Z. These compounds are also known for their highly unpredictable pharmacokinetics and pharmacodynamics from patient to patient, their narrow therapeutic range, as well as for their numerous interactions with food and drugs [33, 34]. There is hence a real need for monitoring those treatments in order to ensure their efficacy and to minimize hemorrhagic complications.

#### Prothrombin time

The PT has been widely used to monitor patients under VKA. It is based on adding thromboplastin, a substitute of endogenous tissue factor (TF), and calcium to citrated decalcified platelet poor plasma (PPP), in order to generate fibrin clot formation [35]. Various factors are to be considered when interpreting results of PT, such as the composition of thromboplastin and the coagulometer (optical or mechanical detection) used for its determination. Thromboplastin reagents are usually made of TF, phospholipids, calcium, and often contain an inhibitor of heparin such as polybrene. The two most common sources of TF are rabbit brain and human recombinant preparations. Lupus anticoagulant or hematocrit may also influence the PT. When introducing a novel thromboplastin reagent made of relipidated tissue factor, responsiveness to lupus anticoagulants should be tested prior to monitor patients under VKA with this reagent.

Before 1980, clotting time was usually expressed in seconds or as a ratio compared to a reference value. This way of expressing results didn’t enable to compare results obtained from different laboratories or determined with different reagents or coagulometers. In 1983, the World Health Organization (WHO) developed the International Normalized Ratio (INR) to standardize the expression of PT for patients under VKA. This way of expressing PT is based on determining the International Sensitivity Index (ISI) of the laboratory thromboplastin compared to an International Reference Preparation (IRP) for which the responsiveness to VKA is known. Depending on the origin of the tissue factor, various IRP are available such as WHO human IRP rTF/95 or ECAA (European Concerted Action on Anticoagulation) rabbit reference reagent EUTHR-1 [36]. Previously, TF was extracted from tissues such as rabbit brain or bovine extracts but nowadays, recombinant human thromboplastins with international sensitivity index (ISI) close to 1 have been designed and are replacing progressively animal reagents.

$$\begin{array}{c} INR={\left(\frac{PT\;\mathrm{patient}}{\mathrm{MNPT}}\right)}^{ISI}\\ \mathrm{Log}\;INR=ISI\;\left(\mathrm{Log}\;PT\;\mathrm{ratio}\right) \end{array}$$

Because of the numerous variables to be considered when determining INR, each laboratory should determine its own ISI locally. However, this procedure of local determination of ISI is quite labor intensive and time consuming. It requires 60 patients stable under VKA and 20 healthy subjects. The reference technique is a manual one. The ISI determination consists of comparing PT determined with IRP on manual technique to PT determined by local coagulometer. Mean normal prothrombin time (MNPT) corresponds to the geometric mean of the 20 healthy subjects. Because of the difficulty of the procedure (e.g. the need for large numbers of normal and patients’ blood samples and the availability of reference thromboplastins), ISI calibration is now rarely performed at local hospital levels. Therefore, commercial calibrators with certified INR have been released by manufacturers in order to simplify the procedure and to validate the local ISI. However, manufacturers’ ISIs and INRs may not reflect local values as, for example, coagulometer calibration ISIs are required and INRs often vary between coagulometers even of the same model and manufacturer used in the same laboratory [37]. Results with VKAs are expected to be further improved by two recent European Action on Anticoagulation (EAA) developments in routine oral anticoagulant control (i.e. simplified local INR derivation with the PT/INR line and prediction of further clinical events by a type of variance growth rate analysis), as demonstrated by a recent EAA multicenter study [38]. In the PT/INR Line method, local PT is plotted against 5 certified INR for plasma calibrators and ISI is then determined using the orthogonal regression [39]. Calibrators used for this determination may be of two different types: lyophilized or frozen plasma prepared from native patient plasmas or plasmas prepared by artificial depletion (selective adsorption) of vitamin K dependent clotting factors. Those two types of calibrators aren’t commutable due to different results [40]. The European Society of Cardiology (ESC) Task Force on Anticoagulants has recently stated that PT/INR Line achieves reliable INR without the need for local ISI calibrations and thus recommended it. The EAA PT/INR Line test plasmas are now available in a five-plasma kit [28].

Recently, a variable growth rate (VGR) analysis was shown in a EAA report published in 2013 to be of greater value than the previously accepted ‘time in INR range’, in predicting ‘clinical events’ during warfarin treatment, particularly in short term oral anticoagulant [41].

#### Point of care devices

Point of care testing (POCT) has been developed for whole-blood samples in order to permit monitoring in an easier way, less invasive and more convenient for the patient. POCT devices are submitted to the same level of requirement for calibration and control as traditional determination on citrated blood [42]. Practically, accuracy and precision of point of care testing seem to be sufficient and comparable to results obtained in a laboratory setting [43]. Recent study revealed that point-of-care patient self-testing at home achieves high-quality warfarin therapy outside of clinical trials and compares favorably with the results achieved in randomized trials or in anticoagulation clinic settings [44]. A recent meta-analysis show that time in therapeutic range (TTR) increased by 5% for personal self-testing (PST)/ personal self-monitoring (PSM) compared with usual laboratory-based monitoring [45]. In addition, a significant reduction in the rate of thromboembolic complications with PST/PSM was observed but not in the rate of major bleeding or overall mortality compared with usual laboratory-based INR monitoring. The frequency of INR testing with PST/PSM is higher than usual laboratory-based monitoring [46], leading to less cost-effectiveness [47]. Based on the preceding considerations, the 9^th^ Edition of the American College of Chest Physicians (ACCP) on the Antithrombotic Therapy and Prevention of Thrombosis, makes a weak recommendation in favor of PSM (not PST) for patients treated with VKAs who are motivated and can demonstrate competency in self-management strategies, including the POC equipment [48].

#### Frequency of testing

At the start of treatment, several days or weeks may be needed to reach steady state due to particularly long VKA’s onset of action [49]. During this stabilization period, frequent monitoring is recommended to adjust dosing based on INR determination according to validated VKA dosing normograms [50] computer-assisted oral anticoagulant dosage program [51]. In patients beginning VKA therapy, INR monitoring should be started after the initial two or three doses of oral anticoagulation therapy [48]. In the hospital setting, INR monitoring should be performed daily until the therapeutic range has been reached for at least two consecutive days [52]. In outstanding patients, starting their treatment, monitoring may be reduced to once every few days until the patient is stabilized at the therapeutic range [52]. For patients who are receiving a stable dose of oral anticoagulants, previous recommendations mention that monitoring should be performed at an interval of no longer than every 4 weeks (Grade 2C) [34]. More recent recommendations advice intervals between controls may be extended to 12 weeks for patients with optimal adherence to treatment [52–55]. Patients more likely to maintain stable anticoagulation are older (>70 years), have an INR target of 2-3 (versus higher targets), and do not have heart failure [55]. If adjustments to the dose are required, then the cycle of more frequent monitoring should be repeated until a stable dose response can again be achieved [52].

Prompt repeat testing after out-of range INR value is associated with better anticoagulation control (higher TTR) and could be an important part of a quality improvement effort for oral anticoagulation [56]. The optimal recall interval after a high (>4.0) or low (<1.5) INR value is within 7 days, and within 14 days after a mildly high (3.1 to 3.9) or mildly low (1.6 to 1.9) INR value [45]. The 9^th^ ACCP guidelines suggest for patients taking warfarin with previously stable therapeutic INRs presenting with a single out-of-range INR within 0.5 units of the range to continue the current dose and retest the INR within 1 or 2 weeks (Grade 2C) [55]. This suggestion is based on the concept that for patients with previously stable INR control, the single mildly out-of-range INR likely represents random variation and does not warrant a change in VKA dose; too frequent VKA dose adjustments tend to destabilize the INR leading to suboptimal control [50]. Available evidence supports this recommendation for patients presenting with an out of range INR where there is no identifiable change in diet or medications to explain the result. However, this recommendation should not take the place of a thorough patient interview and individualized assessment of the patients risk for bleeding and thromboembolism. A one-time dose adjustment is reasonable in the setting of a temporary, but not ongoing, precipitating factor for an out of range INR. A change in maintenance dose is advisable if a precipitating factor is identified and will continue long term (e.g. a new chronic medication or dietary habit) [55].

Recent studies found that different factors are associate with an INR-stability in long-term management such as age <70 years, the absence of chronic diseases, and male gender while congestive heart failure, diabetes, and a target range for INR ≥3.0 were associated with instability [57, 58]. Therefore, in order to improve TTR, and thereby improve patient outcomes, it is recommended to target the INR of 2.5 and to avoid the explicit or implicit pursuit of non-standard INR targets [59]. However, as discussed above, a VGR analysis was shown to be of greater value than the TTR in predicting clinical events [38, 41].

Dietary consumption of vitamin K is also a factor that influences the stability of the INR in patients treated by VKA. Several studies had been performed to assess to benefit of the supplementation in vitamin K in unstable patients and it seems that vitamin K supplement improved the stability of anticoagulant therapy [60–68]. Finally, for patients with INR 4.5 to 10.0 and no symptoms of bleeding, it’s recommended to skip 1 to 3 doses of VKA and retest INR. For patients with INR >10.0, give 2.5 mg oral vitamin K and retest INR next day [50].

#### Interpretation

Several therapeutic ranges have been proposed to assess the therapeutic effects of VKA depending on the clinical indications [28]. There is a significant increase in bleeding risk for INR over 4.5 and thrombotic complications should be considered for INR lower than 2.0 [28, 69, 70]. In clinical studies, one should pay attention to the reliability of the INR determination. For example, in the pivotal trials comparing NOACs with warfarin, evidence of the validation of the stated INR was not provided. In RE-LY two important assessments of INR control (i.e. local ISI calibration and external quality control of INR) were not reported. This “claimed INR” makes cross-trial comparisons difficult [28, 71]. In addition, Poller *et al.* hypothesized that this may be one of the reasons explaining why the EAA patients receiving warfarin suffered considerably less thrombotic and bleeding episodes [38].

### Heparins

#### Unfractionated heparin

The anticoagulant response of treatment doses of heparin is highly variable [72] due to competition of a variable number of plasma proteins with AT for heparin binding and complex kinetics of heparin clearance. Thus, the peak activity and duration of effect increase disproportionately with increasing therapeutic doses (apparent half-life: 30 to 150 min) [73]. Thus, UFH therapy is monitored and the dose is adjusted based on assay results. However, some studies have indicated that monitoring of therapeutic UFH in the treatment of VTE may not always be needed. Unmonitored, weight-adjusted subcutaneous heparin was found to be as safe and effective as weight-adjusted LMWH in a randomized trial of patients with VTE, suggesting that aPTT monitoring of subcutaneous heparin may not be needed [74]. The 9^th^ edition of the ACCP guidelines suggests that, for outpatients with VTE treated with subcutaneous UFH, weight-adjusted dosing should be used without monitoring rather than fixed or weight adjusted dosing with monitoring [48]. In addition, a recent retrospective study has shown that routine monitoring and heparin dose adjustment may be unnecessary for patients receiving doses of at least 30 000 units/day [75], as for these patients, the mean proportion of time with an aPTT of 0.2 anti-Xa IU/mL was 92%. The monitoring is also performed to prevent bleeding but its utility is still controversial [76].

#### Global coagulation tests

##### Activated partial thromboplastin time

The most common assay used to monitor heparin is the aPTT. Based one prospective study performed in 1972 [77], an aPTT ratio (reported therapeutic aPTT range divided by the control value for the reagent) of 1.5 to 2.5 was adopted as the therapeutic range for UFH. However, the definition of the control value is not well established. The ACCP recommends against the use of a fixed aPTT target in seconds for any therapeutic indication of UFH [73, 78, 79]. Each laboratory should determine this reference aPTT ratio range for each combination instrument/reagent and for each lot of their cephalin. A French study has recently shown a 3 to 8 fold aPTT increase for an anti-Xa activity of 0.7 IU/mL (Table 1) [80]. Too sensitive reagents do not allow a precise chronometric measurement and therefore should not be used for UFH monitoring [81, 82]. In addition, mechanical end point coagulometers showed greater sensitivity than optical ones [83].

**Table 1:** **Key points about monitoring of unfractionated heparin, low molecular weight heparins and fondaparinux**[78, 162]

	Indications	Posology and route of administration	Delay for blood sampling	Anti-Xa activity (IU/mL)	aPTT
**Unfractionated heparin**					
**Sodium heparin**	-Prevention of clotting during hemodialysis	Bolus of 1,000 - 5,000 IU followed by 1,000 - 2,000 IU per hour	The sampling is performed whatever the time in case of IV perfusion, preferably 4 to 6h after each dosage variation.	0.3 to 0.7	1.5 to 3.0 – 8.0 the upper limit of normal depending on the reagent
	-Cardiopulmonary bypass	300 units/kg intravenously, adjusted thereafter to maintain the activated clotting time (ACT) in the range 300-400 seconds			
**Calcium heparin**	-Prevention of clotting during hemodialysis	Loading dose of 1,000-5,000 units followed by 1,000-2,000 units/hour	Part-time between 2 injections (6h after injection for a 2 injections/day) or 4h after injection for a 3 injections/day		
	-Cardiopulmonary bypass	300 units/kg intravenously, adjusted thereafter to maintain the activated clotting time (ACT) in the range 300-400 seconds			
**Low molecular weight heparins: 2 injections per day†**					
Enoxaparin	-DVT associated with or not PE	100 IU/kg/12 hours or 1mg/kg/12 hours - subcutaneous	3 to 4 hours after the injection	1.2 (+- 0.17) IU/mL	Slightly prolonged
	-Acute coronary syndrome				
Dalteparin	-Constituted DVT	100 to 120 IU/kg/12 hours – subcutaneous		0.6 (+- 0.25) IU/mL (overdose threshold 1.0 IU/mL)	
Nadroparin	-Unstable angina -Myocardial infarction without Q wave	85 IU/kg/12 hours		1.0 (+- 0.2) IU/mL	
**Low molecular weight heparins: 1 injection per day†**					
Tinzaparin	-Constituted DVT	175 IU/kg/24h	4 to 6 hours after the injection	0.87 (+- 0.15) IU/mL (overdose threshold: <1.5 IU/mL)	Prolonged
	-PE				
Nadroparin	-Constituted DVT	171 IU/kg/24h		1.34 (+- 0.15) IU/mL (overdose threshold: <1.8 IU/mL)	Slightly prolonged
**Fondaparinux**					
Fondaparinux	-Constituted DVT	In patients with DVT or PE, dosing was determined by patient weight, with either 5 mg (weight <50 kg), 7.5 mg (weight 50–100 kg), or 10 mg (weight >100 kg) administered/24hours.	2 to 3 hours after administration	The mean peak steady state concentrations for were 1.20–1.26 mg/L	Not prolonged
	-PE				
	-Acute coronary syndrome	2.5 mg/24 hours	2 to 3 hours after administration	Healthy males receiving a single 2.5 mg dose of fondaparinux had an average peak steady state (3 hours) concentration of 0.39–0.5 mg/L	

†In neonates or children receiving therapeutic LMWH either once or twice daily the drug should be monitored to a target anti-Xa of 0.5–1.0 IU/mL in a sample taken 4–6 hours or 0.5–0.8 IU/mL in a sample taken 2–6 hours after subcutaneous injection [157].

Similar reagent/instrument combinations showed less variation in aPTT results than unlike combinations [82]. Using the same instrument model and same reagent lot but performed in different laboratories, significant statistical and clinical differences in the heparin therapeutic range values are found, owing to variation in the individual plasma samples as well as pre-analytical and analytical variables that can vary greatly between hospitals. It is thus unacceptable for a large hospital network to determine the therapeutic range of heparins at only one institution for the whole network [84]. In addition, the procedure of definition of therapeutic range is not defined and debated [85–87]. In the study that established the therapeutic range using the aPTT ratio, the range of aPTT ratios of 1.5-2.5 corresponds to a heparin’s level of 0.3-0.7 IU/mL as determined by anti-Xa assay [88]. Thus, the more accurate method to determine the aPTT ratios equivalent to 0.3-0.7 is to measure aPTT ratio and anti-Xa activity of patient plasmas treated with different levels of anti-Xa. Spiking a normal pool plasma with heparin solutions at different concentrations doesn’t take the *in vivo* heparin metabolism into account and leads to a more prolonged aPTT in comparison to those of treated patients. The regression relationship is then used to derive the range of aPTT ratios equivalent to 0.3 to 0.7 IU/mL anti-Xa. However, this calibration method may not enhance inter-laboratory agreement in UFH monitoring [89] and it should be noted that the evidence linking these plasma heparin levels to the occurrence of bleeding or thrombosis is of low quality [48].

##### Activated clotting time (ACT)

Activated clotting time is used to monitor higher doses of UFH given to patients undergoing percutaneous coronary intervention (PCI) or cardiopulmonary bypass surgery, because at such higher doses the aPTT becomes prolonged to the point of becoming unmeasurable and unreliable. However, PCI and cardiopulmonary bypass surgery induce major hemostatic abnormalities [90–102]. The target ACT was determined by historical papers in 1955 and 1978 [103, 104]. The clinical relevance of this target ACT is doubtful because it has never been validated in prospective studies and because ACT reagents and instruments have changed over years. The ideal management of oral anticoagulation during cardiopulmonary bypass [105, 106] and catheter ablation for AF [107–109] is still controversial with a wide range of procedures available. During AF ablation, it’s now recommended to achieve and maintain an ACT of 300 to 400 seconds in order to reduce the risk of systemic thromboembolism [110]. However, the ACT is affected by a lot of pre-analytical [111] and analytical variables [112, 113]. Finally, target ACT should be re-determined for the peri-procedural use of NOACs for AF ablation. The management of anticoagulation in adults and older children cannot be extrapolated to neonates, due to physiological differences in hemostasis and the dilutional effects of cardiopulmonary bypass in infants [114].

#### Specific coagulation tests

##### Chromogenic anti-Xa assays

Monitoring of UFH may also be performed by anti-Xa activity measurement. The UFH anti-Xa assay is based on the ability of heparin to accelerate inhibition of a standard concentration of FXa in the presence of antithrombin (AT). The test is performed by diluting plasma in buffer, which may or may not contain exogenous AT or dextran sulfate, and incubating with a specific concentration of FXa. Assays that add exogenous AT or dextran sulphate may overestimate the actual *in vivo* activity of UFH, LMWH or fondaparinux in patients with excess plasma proteins or deficient levels of AT [85, 115]. The phenomenon is also encountered in neonates since antithrombin function is significantly decreased in neonates, and by supplementing the assay with exogenous antithrombin there is a direct disturbance of the physiological scenario [116]. The advantage of anti-Xa activity over aPTT is to not be influenced by variation of inflammatory proteins like factor VIII or fibrinogen, by factor deficiencies and lupus anticoagulant. The anti-Xa activity is also preferred to aPTT in children less than 1-year-old [117], in case of prolonged aPTT before treatment initiation and for patients with important inflammatory syndrome affecting aPTT [85]. When the baseline prolongation of aPTT is due to lupus anticoagulant, an insensitive reagent (giving a normal baseline aPTT) should be used [29]. Qualitative or quantitative AT deficiency should evoke a biological or clinical heparin resistance, with an abnormally short aPTT and a weak anti-Xa activity (when measured by a method without *in vitro* addition of antithrombin). There are differences between commercially available methods but the clinical relevance seems to be limited [85].

The frequency of testing and the therapeutic range are mentioned in Table 1[80].

Finally, a recent large retrospective cohort analysis has shown that patients with disproportionate prolongation of aPTT relative to anti-Xa activity did have a highest 30-day mortality and a highest risk of bleeding. If these data are confirmed prospectively, it may be useful to measure both aPTT and anti-Xa [118].

### Low molecular weight heparins

Low molecular weight heparins show a more predictable anticoagulant response than UFH because the shorter heparin chains exhibit lowered affinity for heparin binding proteins in the plasma. Moreover, thanks to reduced binding to the endothelium, LMWHs have a longer half-life than UFH, and the half-life is dose-independent. LMWHs with longer chain lengths have shorter half-lives than LMWHs with shorter chain length, and therefore are less prone to accumulation. LMWHs are cleared by the kidneys and therefore, can accumulate in the plasma of patients with impaired renal function. Typically, LMWHs are given in fixed- or weight-adjusted doses without monitoring. ACCP guidelines recommend against routine coagulation monitoring (grade 1C) [88]. Indeed, data on the correlation between anti-Xa levels and bleeding risk are controversial [78]. A randomized controlled trial comparing monitored versus unmonitored dalteparin therapy for the treatment of VTE showed no benefit of monitoring [119]. In addition, routine monitoring of anti-Xa levels is costly and inconvenient for physicians, patients and laboratory.

LMWHs may produce some prolongation of the aPTT (from 0.6 IU/mL of LMWH [81]), but their effect on the aPTT is less than that of UFH. Thus, aPTT cannot be used for monitoring [73]. Therefore and accordingly, the measurement of the anti-Xa activity is the recommended test [73]. Recommendations advice to monitor the intensity of anticoagulation via the measurement of peak anti-Xa activity levels with various target ranges depending on the LMWH preparation and the frequency of dosing (Table 1) [78, 120]. One limitation is that thresholds have not always been validated in terms of clinical outcomes [80].

Since every LMWH is different, LMWHs monitoring requires calibration towards the specific LMWH used for therapy [29]. Other limitations of anti-Xa activity measurement include a poor comparability between commercially available anti-Xa chromogenic assays [121, 122], substantial inter-laboratory variation in results [81] and poor correlation to antithrombotic efficacy [123]. In contrast to what is generally assumed, the inter-individual variation of the in vitro pharmacodynamics response is equally higher for UFH and any LMWH, (i.e. 25%) when measured by a global assay like thrombin generation assay [124, 125].

Thus, monitoring may be used in obese patients, in those with renal insufficiency or with cirrhosis [126], when therapeutic doses of LMWH are required during pregnancy and in neonates and infants [102].

### Cirrhosis

The anti-Xa assay cannot be used in patients with liver disease to monitor AT-dependent anticoagulant drugs as it underestimated drug levels [126–129]. This underestimation is due to the acquired AT deficiency in these patients [130]. The addition of exogenous AT corrects the drug level. Dose escalations suggested by a low anti-Xa level will potentially lead to a substantial bleeding risk [126]. Clinical trials on the monitoring, efficacy and safety of heparins are urgently required to improve antithrombotic therapy in patients with cirrhosis.

### Pregnancy

The usefulness to monitor the intensity of therapeutic anticoagulant with LMWH during pregnancy is still controversial. Recommendations vary significantly among recent guidelines [131–134]. For a given dose of LMWH, anti-Xa levels are lower in pregnancy than in the non-pregnant state. Lower levels of anti-Xa levels in pregnant patients receiving therapeutic doses of tinzaparin are observed later in gestation [135, 136]. These observations suggest that higher doses or more frequent dosing may be required to achieve a desired anticoagulant effect among pregnant women. A recent single centre prospective case series of pregnant women requiring anticoagulation (tinzaparin at a daily dose of 175 IU/kg) therapy during pregnancy has shown that weight based anticoagulant therapy did not achieve the target range of anticoagulation throughout pregnancy with more than 50% patients showing subtherapeutic levels. Thus it does not seem that adjusting doses for increasing pregnancy weight is sufficient [137]. Further studies in this field in urgently are required.

### Obese patients

Obesity is an important risk factor for venous thromboembolism [138]. Standard fixed doses are suboptimal in obese patients [139–141]. Thus, ACCP guidelines recommend weight-based dosing in obese patients receiving LMWH prophylaxis or treatment (grade 2C) [88].

In a meta-analysis that included data on 921 patients with a BMI >30, there was no excess in the rate of major bleeding over that observed in non-obese patients who received LMWH in doses adjusted by total body weight [78]. For thromboprophylaxis with fixed-dose enoxaparin and nadroparin, there is a strong negative correlation between total body weight and anti-Xa levels in obese patients [78, 139]. In contrast, prescribing approximately 0.5 mg/kg of enoxaparin daily results in anti-Xa levels that are within or near target levels [142]. In a recent large prospective study on 3928 morbidly obese inpatients, high-dose thromboprophylaxis approximately halved the odds of symptomatic VTE, with no increased risk of bleeding [143]. In conclusion, further studies regarding optimal doses for obese patients with anti-Xa factor measurements are still required.

### Several renal insufficiency

Appropriate dosing of LMWHs in patients with renal insufficiency is less clear. There is an inverse relationship between CR_CL_ and anti-Xa levels [78, 144, 145] and the risk of bleeding complications with LMWHs is higher in patients with impaired renal function [78, 146, 147]. Severe renal insufficiency (CR_CL_ lower than 30 mL/min) is a contra-indication of randomized controlled trials evaluating efficacy and safety of LMWHs. In such patients, UFH is, in most cases, a better choice than LMWHs despite numerous drawbacks [148], as UFH is less dependent on renal function. The data on accumulation with LMWHs other than enoxaparin is limited. When used in full therapeutic doses, nadroparin and dalteparin clearance, but not tinzaparin clearance, was shown to be correlated with Cr_CL_[148–151]. The apparent difference in tinzaparin clearance in patients with severe renal insufficiency may reflect metabolism by hepatic mechanisms, possibly due to the higher molecular weight of tinzaparin compared with other LMWHs. Two approaches are considered to optimize the use of LMWHs in the elderly: anti-Xa monitoring or empiric LMWHs dose reduction. However, it is still debated whether there is a clear benefit in anti-Xa monitoring regarding LMWHs efficacy and safety outcomes, especially in patients with renal impairment [120, 152, 153]. Alternatively, empirically reducing the dose to 50 % of the recommended dose has also been proposed by ACCP with a low grade of recommendation for enoxaparin in patients with ACS or VTE with severe renal impairment [78]. However, the empirical reduction of the initial enoxaparin dose without systematic monitoring could lead to an anti-Xa peak level below 0.5 IU/mL, leading to an increase of the thrombotic risk [154]. No specific recommendations have been made for other LMWH preparations given the lack of sufficient data [78, 155]. When given in prophylactic doses, LMWHs has not been shown to increase the risk of bleeding complications, irrespective of the degree of impairment of renal function [78].

### Neonates and infants

The variability in age-related pharmacokinetic parameter estimates (clearance, volume of distribution and half-life) leads to a different pharmacodynamics profile for anticoagulants in children in comparing to adults [116, 156]. The 9^th^ edition of the ACCP guidelines recommend that in neonates or children receiving therapeutic LMWHs either once or twice daily the drug should be monitored to a target anti-Xa of 0.5–1.0 IU/mL in a sample taken 4–6 hours or 0.5–0.8 IU/mL in a sample taken 2–6 hours after subcutaneous injection [157]. There is a need for robust pharmacodynamics models in pediatric practice. The current recommendations regarding anticoagulant dosing or laboratory monitoring in children are simply extrapolated from adult evidence and are not based on appropriately robust levels of evidence [156]. Therapeutic ranges are not well correlated with clinical outcomes and assays are not standardized. In 2012, a position paper from the Perinatal and paediatric haemostasis subcommittee of the scientific and standardization Committee of the International Society on Thrombosis and Haemostasis (ISTH), recommends a step-wise approach to the generation of this evidence [146]. A recent study has shown that enoxaparin dose titration to achieve therapeutic anti-Xa levels may be affected by assay variability. Attempts to titrate to target anti-Xa values may result in significant dose variation that may or may not benefit pediatric patient care. Neonates or children with normal renal function may be safely treated with weight-based age-appropriate standard dosing without monitoring. Therefore, these authors suggest that a prospective, multicenter, randomized clinical trial comparing the safety and efficacy of exonaparin weight-based dosing with and without anti-Xa dose titration using an anti-Xa standardized assay, is required [158].

### Fondaparinux

Fondaparinux is cleared only by renal function. Biological monitoring is not recommended. Anti-IIa assay and aPTT are not recommended as they have only a very low sensitivity to fondaparinux [159–161]. Anti-Xa measurement should be performed with appropriate calibration allowing results expression in ng/mL. Dose tailoring is not recommended according to anti-Xa results. The target ranges (2 to 3 days after injection) are in mean 1.41 mg/mL (0.97-1.92 for 5^th^ and 95^th^ percentiles). The through values are in mean 0.52 mg/mL (0.24-0.95 for the 5^th^ and 95^th^ percentile) for a patient receiving 7.5 mg once a day [162]. No specific data have been published in the very elderly receiving fondaparinux at curative dose [163]. Healthy males receiving a single 2.5 mg dose of fondaparinux had an average peak steady state (3 hours) concentration of 0.39–0.5 mg/L [164]. In patients with deep vein thrombosis or pulmonary embolism, dosing was determined by patient weight, with either 5 mg (weight <50 kg), 7.5 mg (weight 50–100 kg), or 10 mg (weight >100 kg) administered. The mean peak steady state concentrations for all three-weight classes were 1.20–1.26 mg/L [164, 165].

### Non-vka oral anticoagulants

Non-VKA Oral Anticoagulants (NOACs) have been developed to counter one of the main disadvantages of VKA treatment: the requirement of regular monitoring. However, even if these treatments are given without dose adjustments, several situations or populations may require an assessment of the intensity of anticoagulation (See the “Summary of patients/situations that could require a drug tailoring” section). Moreover, a recent investigation made by the BMJ revealed that the marketing authorization holder of dabigatran etexilate, marketed under the brand name of Pradaxa® in Europe, found that if the plasma levels of the drug were measured and the dose was adjusted accordingly major bleeds could be reduced by 30-40% compared with well controlled warfarin [166]. Similar information was also provided in a study evaluating the effect of dabigatran plasma concentrations and patient characteristics on the frequency of ischemic stroke and major bleeding in atrial fibrillation patients in the RE-LY trial [167]. Thus, the “one dose fits all” marketing slogans behind the approval of these drugs proved to be an illusion, for at least one of these compounds. The drug companies of the other NOACs do not yet provide such information. However, the collection, analyses and distribution of similar data are of particular importance since we cannot afford to deprive us of the opportunity to improve the safety/efficacy profile of these drugs by implementing risk minimization measures, if feasible.

#### Summary of patients/situations that could require a drug tailoring

Even if NOACs are presented as having a predictable pharmacodynamics and pharmacokinetics, several patients or situation could require an assessment of the degree of anticoagulation and probably a drug tailoring.

The clinical situations include: recurrence of thrombosis or bleeding, before urgent surgery or procedure (with last administration in the last 24 h or more if CRCL <50mL/min), before fibrinolytic therapy of acute ischemic stroke, in case of bridging therapy, in case of cardioversion and in the setting of dual or triple antithrombotic therapy, such as in the patient with AF undergoing a percutaneous coronary intervention, when dual platelet inhibitors may be added to NOACs, given that such patients represent a complex management problem.

In addition, several patterns in patient status could also require an assessment of the responsiveness at the individual level. This includes patients with risk factors for NOACs accumulation or too low levels (i.e. drug-drug interactions as with frequently used medication like amiodarone and verapamil), patients with extreme body weight (<50 kg or >110 kg), patients with hepatic impairment, patients with renal impairment (in case of progressive decrease of renal function but also in acute decrease during dehydration, antibiotics administration, …), in case of comorbidities or in elderly patients*.*

#### How to accurately measure plasma drug concentrations?

In this part of the manuscript, we review the different routine coagulation tests that could be used to estimate the intensity of anticoagulation in patients treated with dabigatran etexilate (the pro-drug of dabigatran, a direct thrombin inhibitor) and with rivaroxaban or apixaban, two direct factor Xa inhibitors. More specific assays used to accurately estimate plasma drug concentrations are also presented.

### Global coagulation tests

#### Dabigatran: activated Partial Thromboplastin Time

The recent recommendation of the Subcommittee of Control of Anticoagulation of the Scientific and Standardisation Committee of the ISTH, mentions that the aPTT using most available reagents can be used to determine the relative intensity of anticoagulation due to dabigatran. However, they state that aPTT should not be used to quantify the drug plasma concentration. They add that each laboratory should be aware of the sensitivity of their aPTT assays to dabigatran and this can be achieved using commercially available plasma calibrants [168]. However, it is unknown if specific dabigatran calibrants, used out of their dedicated platform context, are truthful calibrants that could reflect accurately the impact of dabigatran in plasma from patient’s sample, since aPTT is affected by numerous pre-analytical and biological variables.

**Figure 1:**
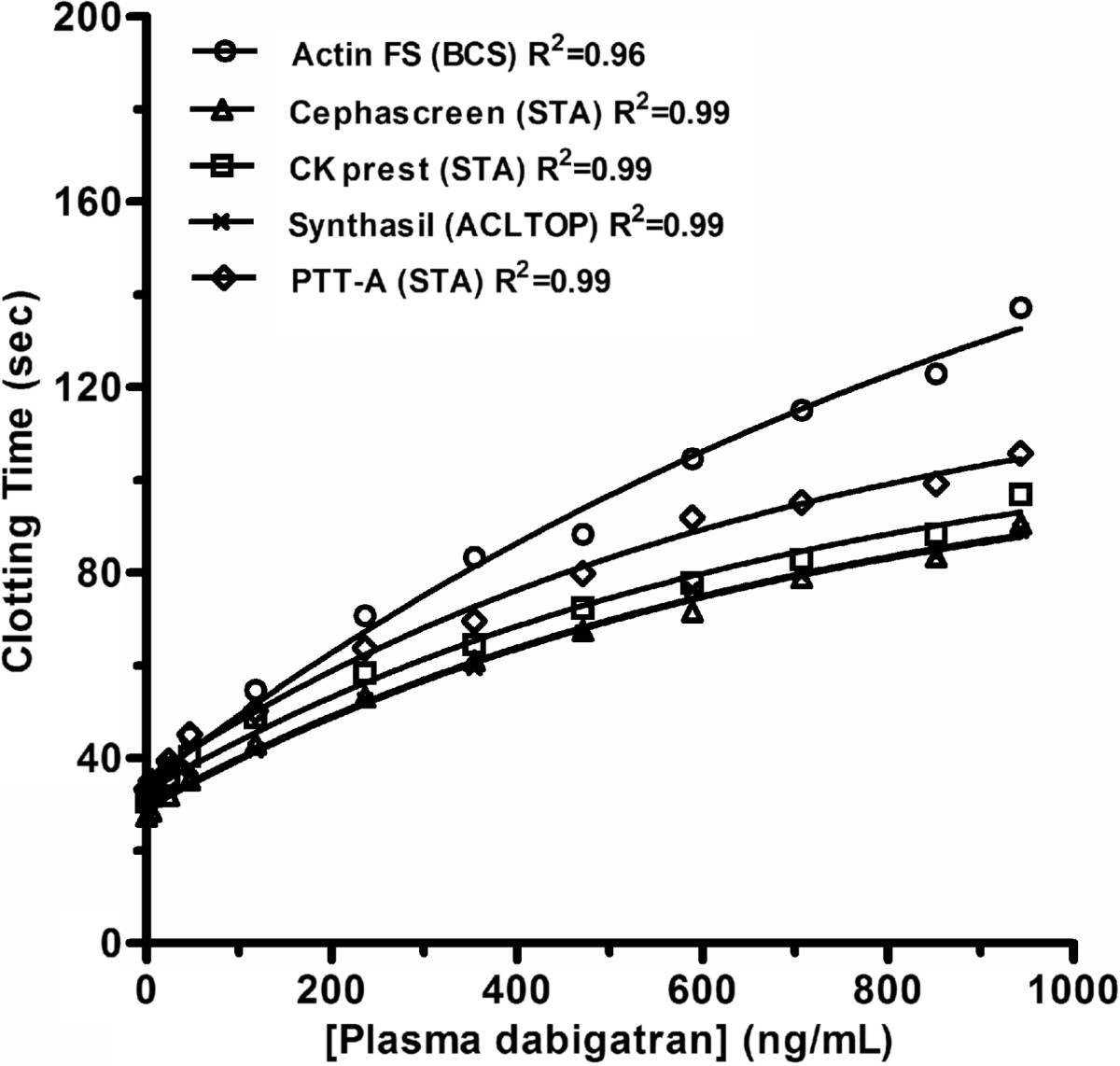
**Impact of dabigatran on several aPTT reagents.**

It is stated in the EU-SmPC that when dabigatran was used for the prevention of stroke in NVAF with a *bid* dosing regimen, an aPTT ratio greater than 2xULN (or an aPTT prolongation of about 80 seconds) at trough (10-16 h after the previous dose) reflected the 90^th^ percentile of observations (i.e. 200 ng/mL at C_trough_) and is considered to be associated with a higher risk of bleeding [169]. However, studies revealed that the inter-reagent variability prevents using an aPTT of about 80 seconds as reflecting plasma dabigatran concentration of 200 ng/mL [170] (Figure 1). Similar observations have been demonstrated for the threshold proposed in VTE prevention regarding the bleeding risk [170]. Moreover, recent findings revealed that in addition to the inter-reagent variability, the different combinations between reagents and coagulometers increased further this variability [171]. Therefore, laboratories should be aware about the sensitivity of their aPTT reagents towards dabigatran assessed with homemade calibrants using local normal pooled plasma spiked with dabigatran.

Thus, aPTT has limited sensitivity depending on the reagent and is not suitable for precise quantification of the anticoagulant effect for several reasons. First, the aPTT is affected by pre-analytical and biological variables [172, 173]. Secondly, a prolonged aPTT is not strongly predictive of hemorrhage and patients may experience bleeding while displaying a normal aPTT [173–175] and finally, the dose-response is not linear, precluding the possibility to differentiate minor versus major overdoses (Figure 1).

##### Rivaroxaban: Prothrombin Time/INR

The Subcommittee of Control of Anticoagulation of the Scientific and Standardization Committee of the ISTH mentions that PT (with a sensitive reagent) can be used to determine the relative intensity of anticoagulation in emergency situation when required, but should not be used to quantify drug plasma concentrations [168]. However, PT results of samples from patients treated with rivaroxaban cannot be translated to INR values since INR was developed to normalize PT in patients treated by VKA thanks to the International Sensitivity Index (ISI) specifically determined for VKA therapy.

**Figure 2:**
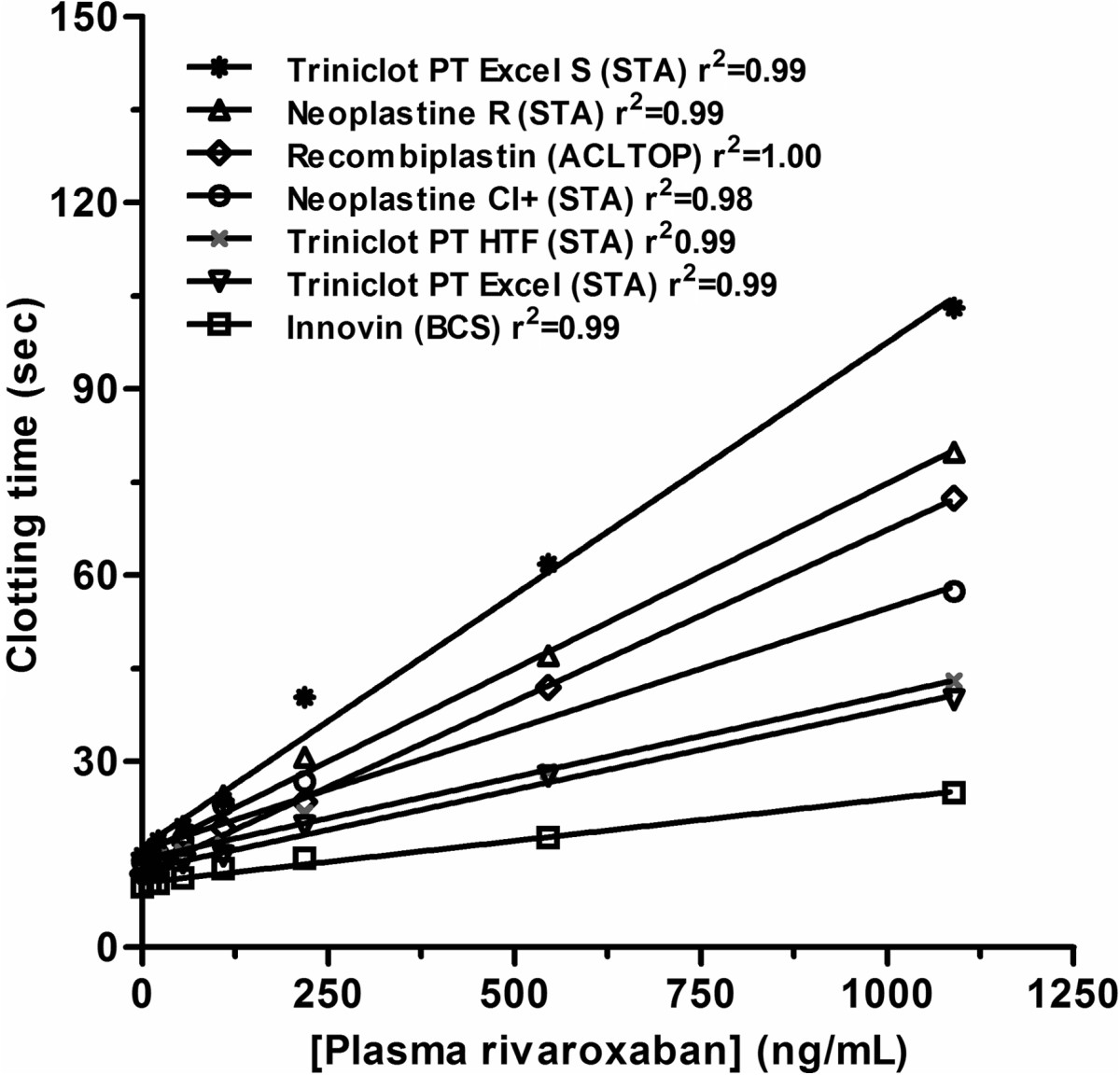
**Impact of rivaroxaban on several PT reagents.**

*In-vitro* studies reported a large PT reagents variability and, as for dabigatran and the aPTT, the different combinations between PT reagents and coagulometers increased further this variability [160, 171, 176–178] suggesting that laboratories should be aware about the sensitivity of their own reagent towards rivaroxaban (Figure 2). The Subcommittee of Control of Anticoagulation of the Scientific and Standardization Committee of the ISTH support this statement [168]. Nevertheless, one weakness of this approach is that commercially available calibrants are labelled to be used with their corresponding chromogenic anti-Xa assays. Therefore, similarly to dabigatran and the aPTT, the quality and the accuracy of these calibrants for the calibration of PT reagents are not warranted. In addition, an *ex-vivo* study revealed a poor correlation between calibrated-PT and measured rivaroxaban plasma concentration [179].

Therefore, depending on the reagent, PT must not be used to estimate rivaroxaban concentrations in plasma and poorly reflects the intensity of anticoagulation due to rivaroxaban. The poor sensitivity, the important variability and the poor linear correlation with the LC-MS/MS in patients’ plasma samples preclude the use of PT to estimate rivaroxaban plasma concentration.

##### Apixaban: Prothrombin Time/INR or modified PT

**Figure 3:**
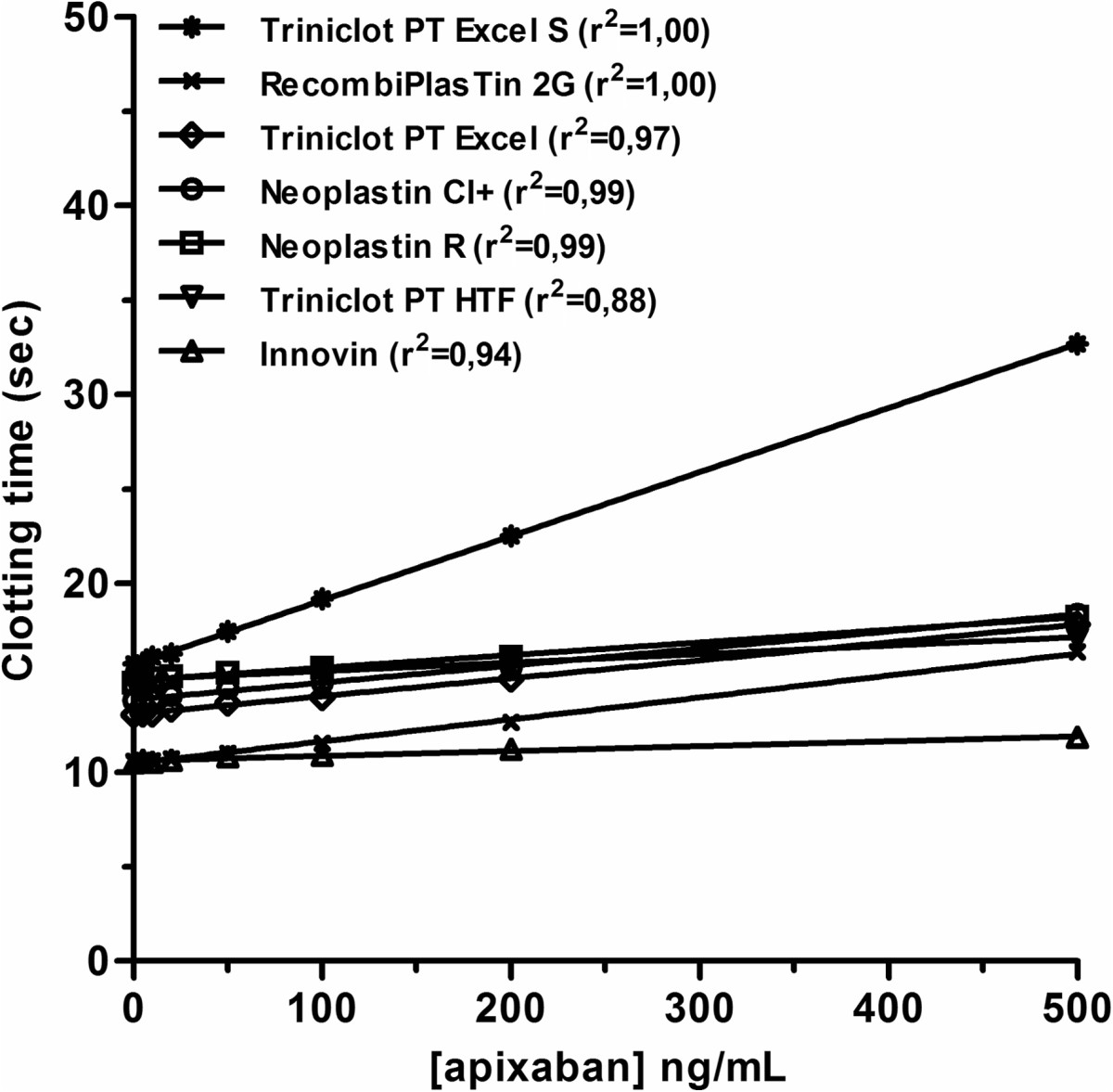
**Impact of apixaban on several PT reagents.**

As stated for rivaroxaban, INR must not be used for the assessment of apixaban while PT, either expressed in seconds or as ratio, is not sensitive enough to ensure an accurate quantitative measurement of apixaban [180–182]. Moreover, depending on the reagent, PT may be normal with therapeutic concentration of the drug [182, 183]. For the most sensitive reagents it may only inform the clinician if the patient is taking the drug. This inter-reagent variability (Figure 3) prevents valid recommendations of cut-offs in seconds associated with a bleeding risk applicable to all reagents [181]. In addition, drugs or hematologic abnormalities affecting at least one factor assessed by PT could bias the conclusions. We definitively do not recommend PT to estimate plasma concentration of apixaban. During the early clinical development of a series of novel factor Xa inhibitors, a modified PT (mPT) assay was developed in which calcium chloride (CaCl_2_) was added to the thromboplastin reagent in order to prolong clotting times and, hence, increase the sensitivity of the dose–response curve for the direct factor Xa inhibitor [184]. Thus, mPT method could be used for the assessment of the pharmacodynamics activity, but the limitations highlighted previously for PT might remain valid and the inter-reagent and inter-individual variability must be assessed. With further development and standardization, this assay could provide a potential option [181, 184].

#### Specific coagulation tests

##### Dabigatran: dilute Thrombin Time (dTT): Ecarin Clotting Time (ECT) and Ecarin Chromogenic Assay (ECA)

Thrombin Time (TT) was demonstrated to be too sensitive towards dabigatran [170, 185] and led to the development of a calibrated diluted thrombin time (dTT) using dabigatran standards to calculate the plasma concentrations. Hence, the CE-marked Hemoclot Thrombin Inhibitor® (HTI) was developed and has been proposed as a rapid, standardised and calibrated assay to determine plasma concentrations of dabigatran [170, 185–187]. The coagulation test is based on the addition of highly purified thrombin in the α-form in plasma samples pre-diluted in physiological serum (1/8 ratio) and normalized with a defined amount of normal pooled plasma. By diluting plasma samples, the test is less sensitive to dabigatran and allows the quantitation of dabigatran concentration from 50 to 500 ng/mL. It is fully automatable and has been adapted to different coagulometers in order to be easily implemented in laboratories. Several studies showed that HTI highly correlates with dabigatran plasma concentrations measured by LC-MS/MS in patient’s plasma [185, 186, 188, 189]. Nevertheless, for the accurate determination of dabigatran plasma concentrations below 50 ng/mL, the more sensitive LC-MS/MS method is still required [186, 188].

The ECT assay provides a direct measure of the activity of direct thrombin inhibitors. Ecarin is a snake venom extracted from *Echis carinatus*. Ecarin cleaves prothrombin to form meizothrombin, an active effector able to transform fibrinogen to fibrin. Meizothrombin is sensitive to direct thrombin inhibitors (DTIs) but is unaffected by heparin and its derivatives as well as by antithrombin [190]. While development of commercial kits may improve the practicality of this test, these kits have not been standardised or validated with dabigatran [185]. For these reasons, ECT cannot be recommended for emergency monitoring of anticoagulant effects. Moreover, ECT is not widely available and is known to have inter-lot variability indicating that calibration is also required with this test [170].

Recently, the ECA, the chromogenic variant of ECT, has been specifically developed to accurately estimate the plasma concentration of dabigatran and other DTIs in plasma. In this test, ecarin converts an excess of exogenous prothrombin added in the diluted plasma sample to form meizothrombin. The cleavage of the chromogenic substrate by the residual meizothrombin released *p-nitroaniline (pNA)* that can be measured at 405nm. The quantity of *pNA* generated is inversely proportional to the quantity of DTIs in the plasma. For dabigatran measurements, the test is calibrated with standard calibrants and provides a lower limit of quantitation similar to the one obtained with HTI. However, this test is not yet approved [188, 189, 191].

##### Rivaroxaban: chromogenic anti-Xa assays

Thanks to specific calibrants and controls containing a defined amount of rivaroxaban, a dedicated chromogenic anti-Xa assay has been proven to accurately estimate plasma rivaroxaban concentrations >30 ng/mL [179]. Several chromogenic anti-Xa assays are available on the market, however, only some of them are labelled to ensure the quantitation of rivaroxaban plasma concentrations. It is therefore important to work on specific coagulation platforms where it was previously found that the mean CV is lower in the inter-laboratory setting [192].

However, taking into account the lower sensitivity of chromogenic assays compared to LC-MS/MS and the variability of coagulation analysers that may further increase the imprecision at the lowest concentrations, detection and quantitation of lower levels (<30 ng/mL) in rivaroxaban treated patients still requires LC-MS/MS analyses [179, 193]. Consequently, the LC-MS/MS is required for quantification of very low to moderate rivaroxaban concentrations (3 to 30 ng/mL) in clinical samples.

##### Apixaban: chromogenic anti-Xa assays

Due to their good sensitivity towards the inhibition of FXa by apixaban, chromogenic anti-Xa assays calibrated with specific apixaban calibrants could estimate plasma drug concentrations [181, 183]. Patients of the APPRAISE-1 study had participated in a PK/PD study suggesting that apixaban-mediated anticoagulant effect can be detected using a standard laboratory chromogenic anti-Xa assay with either LMWH or apixaban calibrants [194]. However, the authors failed to mention that the chromogenic anti-Xa assay tended to underestimate the plasma drug concentration when comparing plasma apixaban concentrations estimated by the calibrated STA®-Rotachrom® and the true plasma concentration measured by LC-MS/MS [194]. Thus, further studies are required with validated calibrants to compare dedicated calibrated chromogenic anti-Xa assays with LC-MS/MS in real-life patients treated by Eliquis®. As for rivaroxaban, it seems to be preferable to work on specific coagulation platforms to reduce the inter-laboratory CV [183, 195].

## Authors’ original submitted files for images

Below are the links to the authors’ original submitted files for images. Authors’ original file for figure 1 Authors’ original file for figure 2 Authors’ original file for figure 3
