# Supplementary figures and images for: Measurement of non-VKA oral anticoagulants versus classic ones: the appropriate use of hemostasis assays
Source: Thromb J. 2014 Nov 4;12:24. doi: 10.1186/1477-9560-12-24 (PMC4351835; doi:10.1186/1477-9560-12-24)

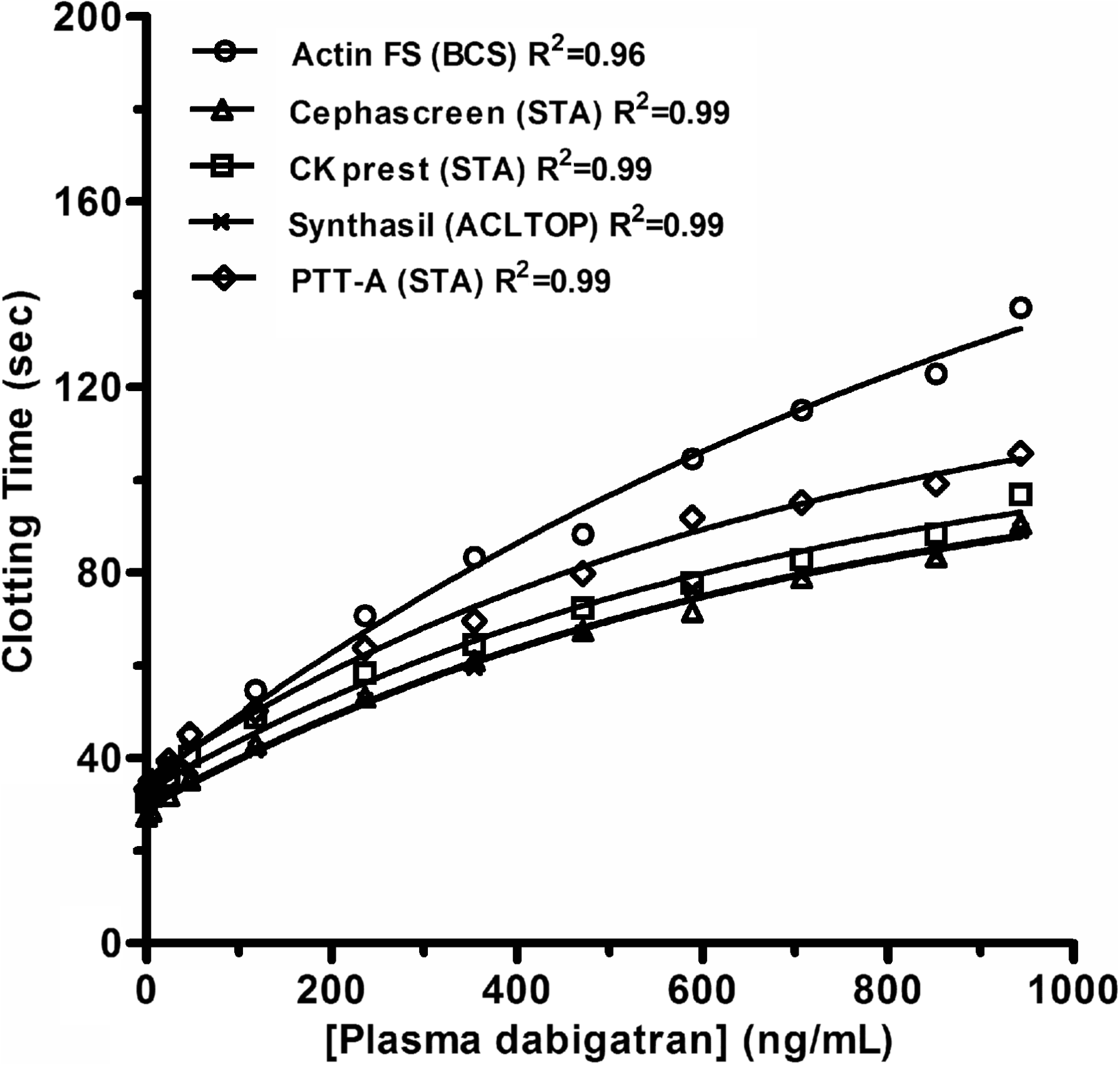

Supplement: Supplementary file 1 — Authors’ original file for figure 1 [file 12959_2014_215_MOESM1_ESM.tif]

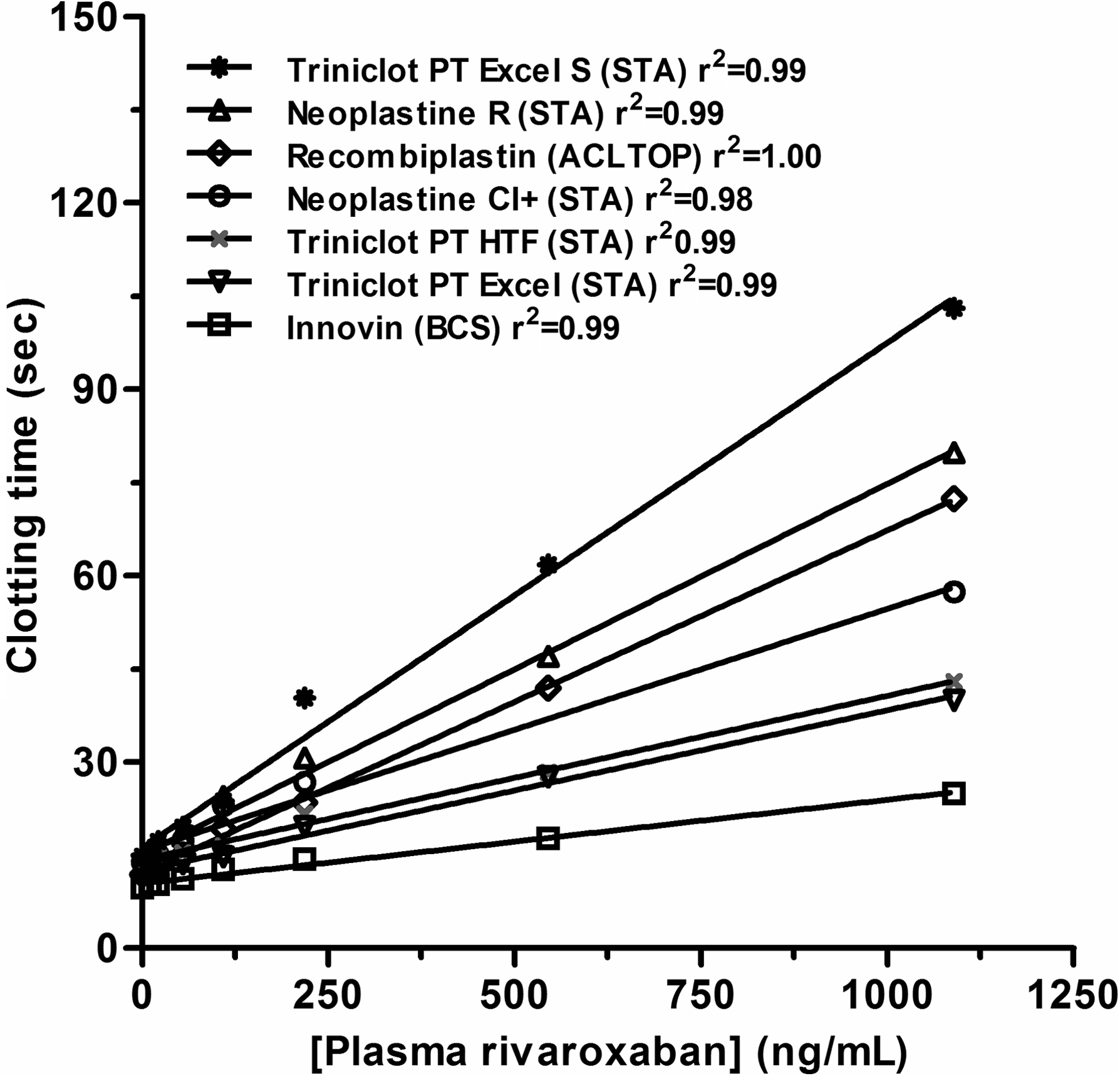

Supplement: Supplementary file 2 — Authors’ original file for figure 2 [file 12959_2014_215_MOESM2_ESM.tif]

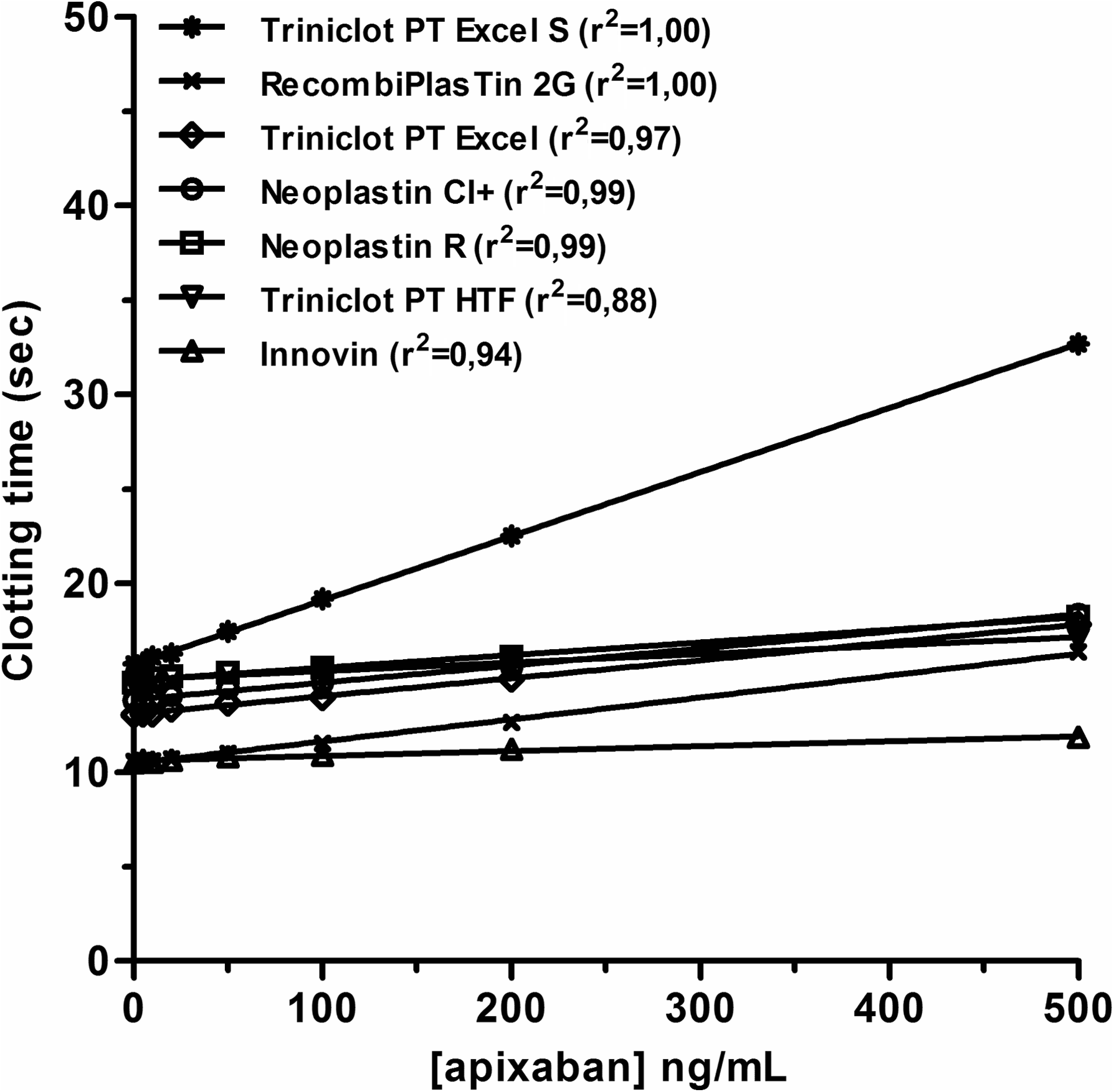

Supplement: Supplementary file 3 — Authors’ original file for figure 3 [file 12959_2014_215_MOESM3_ESM.tif]
